# Obstetrical Work from the Standpoint of the General Practitioner

**Published:** 1908-08

**Authors:** A. B. Croom

**Affiliations:** Maxton, N. C.


### OBSTETRICAL WORK FROM THE STANDPOINT OF THE GENERAL PRACTITIONER.

BY A. B. CROOM, M. D., MAXTON, N. C.

This subject is one which should interest every medical man; for no section is without its share of such work, and to the general practitioner or family physician usually falls these duties.

Obstetrics, we all concede, is a line of our profession entirely distinct and yet very closely connected with the general practice of medicine. Still it is a line of work falling more on the surgical than the medical side.

It is my desire in these brief remarks to emprasize the surgical side and to point out or call attention to what I regard as a few of the most important points to be remembered in attending the usual run of lying-in cases.

1st. Remember that in complicated conditions, when interference is necessary, an experienced and skilled obstetrician is required, and even when we feel ourselves thoroughly competent to do the said skilled piece of work that an assistant should be called.

2nd. Of still more importance is it to remember that Nature is the Great Obstetrician and will in the majority of cases be able to pull the patient through without our "skilled assistance," therefore do not interfere unnecessarily, and do not be guilty of "meddlesome midwifery."

3rd. Remember that from start to finish in any labor case there is nothing of more importance than *asepsis*.

Here I may remark that while I am well aware that some of my friends regard this a matter of choice, or some might say useless and unnecessary, still I wish to lay especial stress upon the use of sterile rubber gloves as a routine. It has been a great satisfection to me in my few years experience to fell sure, when finding a slight rise of temperature, that it could not be any infection coming from improper cleaning of my hands.

It is of course the duty of one who announces himself in this line of work to be ready to meet promptly all emergencies. The obstetrical bag should be kept in thorough readiness. It should contain the usual articles as sterile gauze, cotton, perineal pads.

nail brush, soap, bichloride tablets; and in addition such instruments as forceps, needles, silk or other suture material, etc.

In event of a tear of the perineum or cervix it should be repaired immediately. And before such repairs can be done they first must be found; therefore do not fail to look carefully.

Preparations are made for care of the baby before the rush, and means of resuscitation should be at hand always—hot and cold water, a flannel wrap for the child, etc.

Post partum hemorrhage always should be thought of in time to avoid it. The bladder and rectum should be emptied at the beginning of labor. Quick and vigorous kneading of the uterus, as soon as the second stage is past, should be continued through the third stage and further until the contraction is firm and a hard uterus is felt.

A routine dose of ergot after the placenta is delivered is my practice. It may save trouble and cannot do harm.

The after treatment of these cases is of importance. The duty of the attending obstetrician is by no means complete when his patient is safely delivered and comfortable. Perineal pads should be applied immediately after the patient is cleaned up and before any change of clothing and bedding. These should be used during the entire puerperal period and changed as frequently as indicated—every few hours during the first day or two and thereafter as necessary, two or three times daily. The patient should be kept quiet and comfortable in bed, not remaining in any one position long enough to allow a slight displacement to give trouble in the future. The diet should be regulated largely by the condition of the patient—the breast or rather the milk-supply being the chief index. If the milk is scanty, a liquid diet—cocoa, tea, milk, etc. If abundant and breasts are giving pain a dry diet is indicated.

The normal cases should remain in bed for ten days and should not show the least rise of temperature. The practice of giving a saline or castor oil purge on the second day is a good one and often saves a slight rise of a half or one degree in the temperature. A rise should be regarded with suspicion and if the odor of the lochia indicates the least trouble, especially if we have a chill—intra-uterine douches of hot saline solution should be promptly and frequently employed until all temperature and odor have gone.

